# Comprehensive analysis of 7-methylguanosine and immune microenvironment characteristics in clear cell renal cell carcinomas

**DOI:** 10.3389/fgene.2022.866819

**Published:** 2022-08-08

**Authors:** Yu Xiao, Junfeng Yang, Maolin Yang, Jinjun Len, Yanhong Yu

**Affiliations:** ^1^ The Affiliated Hospital, Kunming University of Science and Technology, Kunming, China; ^2^ Department of Urology, The First People’s Hospital of Yunnan Province, Kunming, YN, China

**Keywords:** clear cell renal cell carcinomas, m7G, tumor immune microenvironment, immunotherapy, immune checkpoints

## Abstract

Clear cell renal cell carcinoma (ccRCC) is one of the most common tumors in the urinary system. ccRCC has obvious immunological characteristics, and the infiltration of immune cells is related to the prognosis of ccRCC. The effect of immune checkpoint therapy is related to the dynamic changes of the tumor immune microenvironment (TIM). The 7-methylguanosine (m7G) is an additional mRNA modification ability besides m6A, which is closely related to the TIM and affects the occurrence and development of tumors. At present, the correlations between m7G and the immune microenvironment, treatment, and prognosis of ccRCC are not clear. As far as we know, there was no study on the relationship between m7G and the immune microenvironment and survival of clear cell renal cell carcinomas. A comprehensive analysis of the correlations between them and the construction of a prognosis model are helpful to improve the treatment strategy. Two different molecular subtypes were identified in 539 ccRCC samples by describing the differences of 29 m7G-related genes. It was found that the clinical features, TIM, and prognosis of ccRCC patients were correlated with the m7G-related genes. We found that there were significant differences in the expression of PD-1, CTLA4, and PD-L1 between high- and low-risk groups. To sum up, m7G-related genes play a potential role in the TIM, treatment, and prognosis of ccRCC. Our results provide new findings for ccRCC and help to improve the immunotherapy strategies and prognosis of patients.

## Introduction

ccRCC is one of the most common tumors in the urinary system, and the incidence of renal cell carcinoma is increasing year by year. About 400,000 people worldwide suffer from renal cell carcinoma every year, accounting for about 2%–3% of all cancers (Siegel et al., 2021; Sung et al., 2021). With the development of endoscopy and the improvement of adjuvant chemotherapy and immunotherapy, the mortality rate has decreased in developed areas (Antonelli et al., 2018; Tao et al., 2021). The successful application of immunotherapy in many kinds of cancer and further study of the tumor immune microenvironment may change our current management mode of patients and help some patients achieve long-term survival and even achieve the goal of clinical cure (Gandara et al., 2018; Mazieres et al., 2021).

Recent epitranscriptomic studies reveal that more than 170 post-transcriptional ribonucleic acid (RNA) modifications can be identified in eukaryotic RNAs. This type of RNA is generally catalyzed by a highly specific and conserved enzyme, and its destruction will lead to a series of diseases (Jonkhout et al., 2017). The most common and widespread mRNA modification in mammals is N6-methyladenosine (m6A) (Boccaletto et al., 2018). The internal modification of transfer RNA (tRNA) affects the stability of its structure and function and has a significant correlation with protein expression (Zheng et al., 2017). Maladjusted tRNA modification affects the occurrence and development of tumors (Rapino et al., 2021). M7G is a conserved modified nucleoside, which is most often located at the 46th position in the variable region of tRNA. M7G is found in eukaryotes and eubacteria, and even in some archaea (Frye et al., 2018). The expression of m7G was still found in the psychrophilic bacteria with very low content of modified nucleosides.

M7G plays an important role in ischemic diseases. Some studies have found that m7G-related gene METTL1 promotes the translation of VEGFA mRNA, which is positively related to post-ischemic angiogenesis (Zhao et al., 2021). Dysregulation of RNA is associated with tumors. Highly expressed m7G-related genes were found in the lung cancer patient tissues, and METTL1 promoted the development and metastasis of lung cancer through the modification of tRNA (Ma et al., 2021). In addition, high expression of METTL1 is often associated with poor prognosis in tumor patients, and METTL1 depletion affects m7G tRNA modification and changes the cell cycle, which is negatively correlated with oncogenicity (Orellana et al., 2021). Similarly, METTL1 is significantly expressed in intrahepatic cholangiocarcinoma, which affects the progression and prognosis of patients. M7G-related genes are closely related to the treatment and progression of tumors, suggesting that it may become a new target of immunotherapy.

At present, the direct correlation between the treatment of ccRCC and m7G is not clear. The occurrence and development of tumor is affected by many factors. Therefore, a multi-angle understanding of the correlations between m7G and TIM is helpful to provide new insights into the treatment of ccRCC. To the best of our knowledge, there is no study of m7G on the survival and immunotherapy response of ccRCC, and our study provides important suggestions for the management of ccRCC patients.

## Materials and methods

### Data sources

RNA sequencing transcriptome profiling harmonized to the fragments per kilobase million (FPKM) of 539 ccRCC samples, 72 normal samples, and corresponding clinical data were downloaded from The Cancer Genome Atlas (TCGA) database (https://portal.gdc.cancer.gov/repository). The simple nucleotide variation (Masked Somatic Mutation) was also downloaded from the TCGA database. The GSE16449 cohort containing 70 ccRCC samples were downloaded from the Gene Expression Omnibus (GEO) (https://www.ncbi.nlm.nih.gov/geo/). The ccRCC copy number data (gene-level) were downloaded from UCSC Xena (https://xena.ucsc.edu/). For the TCGA ccRCC cohort, fragments per kilobase million (FPKM) values were transformed into transcripts per million (TPM) (Conesa et al., 2016). Combining the TPM data of TCGA and GSE16449 data, the normal samples were removed, and the “sva” package of R software was used for batch correction. For datasets in public databases, institutional review board approval and informed consent were not required. The patients’ clinical information is described in Supplementary Table S1.

### Unsupervised cluster analysis

Altogether, 29 m7G-related genes were retrieved from a prior review (Tomikawa, 2018) and the Gene Set Enrichment Analysis (GSEA) website (http://www.gsea-msigdb.org/gsea/index.jsp, Supplementary Table S2). According to the expression of m7G-related gene, we used the “ConsensusClusterPlus” package of R software for unsupervised cluster analysis and divided the patients into different molecular subtypes. We ensured that the intra-group correlation was large and the inter-group correlation was small. We used the “GSVA” package of R software for gene set variation analysis (GSVA) to study the differences in biological processes of the m7G-related gene (Ferreira et al., 2021); the gene set file (c2.cp.kegg.v7.4.symbols.gmt) was obtained from the MSigDB database (https://www.gsea-msigdb.org).

### Correlations between the molecular subtypes and prognosis of clear cell renal cell carcinoma

We compared the correlations between molecular subtypes and clinical characteristics of patients and analyzed the correlations between m7G-related genes, molecular subtypes, and survival of ccRCC patients by “survival” and “survminer” packages of R software (Wang H et al., 2020).

### Correlations of molecular subtypes with the tumor immune microenvironment

We used the single-sample gene set enrichment analysis (ssGSEA) algorithm to determine the relative abundance of immune cell infiltration in TIM (Racle and Gfeller, 2020). The immune score was calculated using the “ESTIMATE” package.

### Differentially expressed genes

We used the “limma” package of R software to obtain DEGs and made the adjusted *p* value < 0.05. We used the “clusterprofiler” package for enrichment analysis of gene ontology (GO), functional annotation, and Kyoto Encyclopedia of Genes and Genomes (KEGG).

### Generation of 7-methylguanosine score

Individual ccRCC patients were evaluated by constructing a m7G scoring system. First, a univariate COX regression analysis was used for DEGs, and unsupervised clustering method was used to divide the patients into different subtypes (m7Gcluster A and B) for a follow-up analysis. Second, the patients were divided into different subtypes (geneCluster A, B, and C) according to the expression of m7G-related genes. Then patients with ccRCC were divided into equal number of train group (*n* = 265) and test group (*n* = 253). Finally, through cross-verification, the optimal LASSO regression model was constructed. The risk score formula was obtained using the coefficient and gene expression level calculated by LASSO regression.

m7G Score=∑(Expi ∗ Coefi). (1)

Here, Expi and Coefi represent the expression level and risk coefficient of each gene, respectively. According to the median risk score, the samples of the test group and the train group were divided into high- and low-risk groups, and subsequent survival analysis was carried out. The ROC curve was depicted, and the area under the curve (AUC) of the test group and train group was calculated for 1, 3, and 5 years with the “timeROC” package of R software. Principal component analysis (PCA) was carried out with the “ggplot2” package of R software.

### Establishment of a nomogram

Based on the patient’s clinical information and risk score, we used the “RMS” package of R software to establish a simple and effective nomogram (Iasonos et al., 2008). Each variable in the figure can get a score, and the sum of all the scores is the final result of the patient. Nomogram is used to describe 1-year, 3-year, and 5-year survival forecasts.

### Correlation between 7-methylguanosine score and immune cells

We evaluated the correlation between seven genes involved in the model and 22 immune cells, analyzed the differential expression between the low-risk group and the high-risk group, and discussed the correlations between the two risk groups and cancer stem cells (CSCs).

### Mutation and immune checkpoint analysis

We used the “maftools” package of R software to evaluate somatic mutations in patients in high-risk and low-risk groups (Mayakonda et al., 2018). We also performed a stratified analysis according to age, sex, TNM stage, and survival status of kidney cancer patients to determine the objective predictive power of the m7G score and analyzed the correlation of the m7G score with immune checkpoints. Finally, we verified the expression of some m7G-related genes and risk score genes in tumor tissue and normal tissue by immunohistochemistry (METTL1, EIF3D, NUDT11, NUDT16, EIF4A1, IFI44, and CYFIP1).

### Statistical analysis

All the statistical analyses and picture drawings were carried out using the R software (version 4.1.2). *p* < 0.05 was considered to have the statistical difference; the Benjamini–Hochberg (BH) multiple test correction was used to calculate the adjusted *p* value.

## Results

### Variation and expression of 7-methylguanosine-related genes in clear cell renal cell carcinoma

**FIGURE 1:**
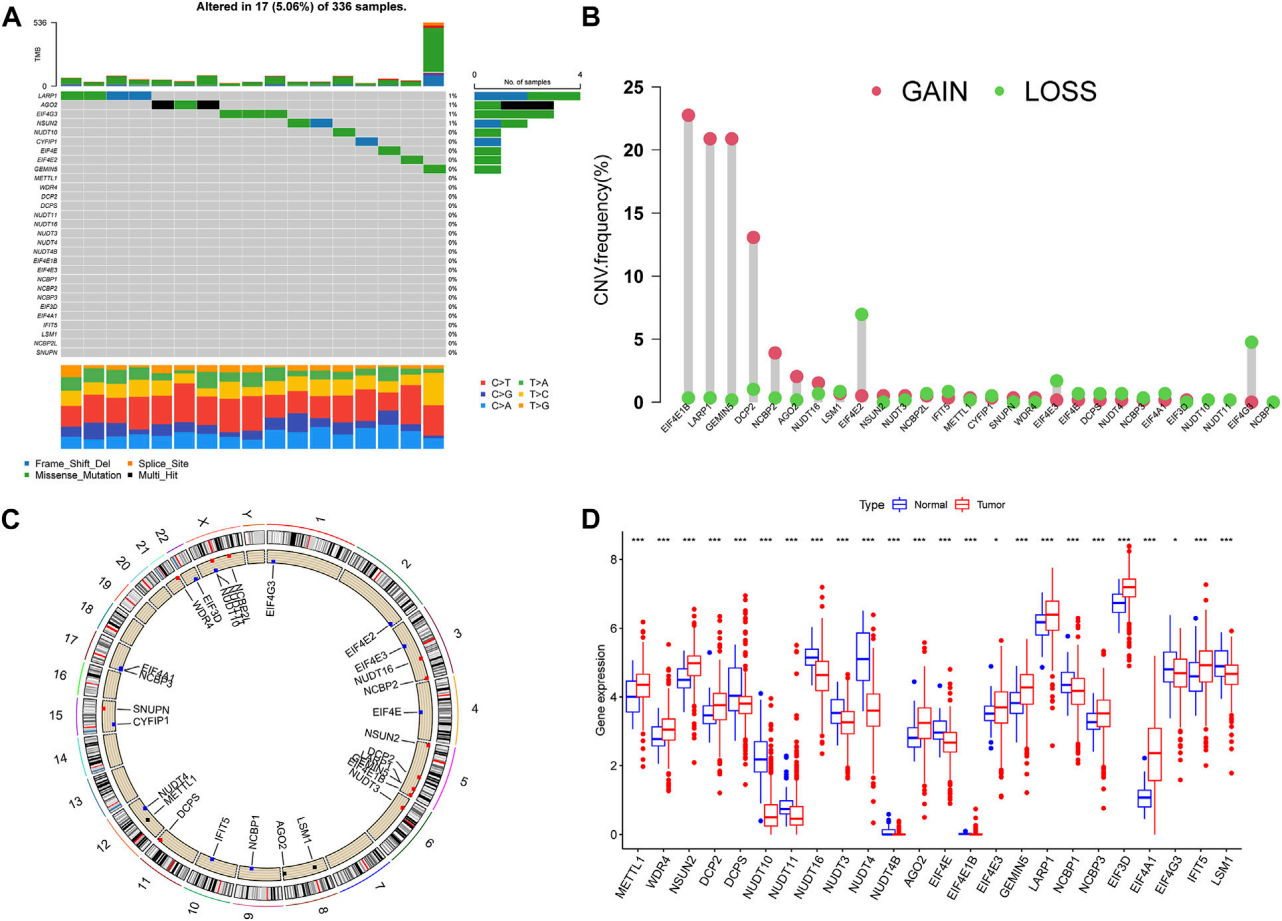
Variation of m7G-related genes in ccRCC. **(A)** Genetic alteration on a query of m7G-related genes. **(B)** The frequency of CNV in m7G-related genes. **(C)** The location of the CNV alteration of the m7G-related genes changes on 23 chromosomes. **(D)** Gene expression of m7G-related genes in ccRCC and normal samples. *p < 0.05, **p < 0.01, ***p < 0.001; ccRCC, clear cell renal cell carcinoma.

We studied the incidence of somatic mutation in 29 m7G-related genes (Figure 1A). There were 17 sample mutations in 336 samples, with a mutation rate of 5.06%. Among them, the mutation rate of LARP1 was the highest, followed by AGO2, EIF4G3, and NSUN2. Overall, the m7G-related gene mutation rate was not high in ccRCC; there were 20 m7G-related genes without mutation in any sample. Through the analysis of copy number variation (CNV) of m7G-related genes, it was found that with the exception of NUDT4B, all 28 m7G-related genes had copy number variation. EIF4E1B had the most significant copy number variation, followed by LARP1, GEMIN5, and DCP2, and the most significant copy number deletion was EIF4E2 and EIF4G3 (Figure 1B). Next, we observed the location of m7G-related genes copy number variation on chromosomes (Figure 1C). And we further evaluated the expression of m7G-related genes in normal samples and ccRCC samples. In the two groups, most of the m7G-related genes’ expression was significantly different (Figure 1D). We observed a high expression of METTL1 in tumor samples, which is consistent with the conclusions of previous studies. The expression of EIF3D is the highest in tumor samples. Some studies have shown that the expression of EIF3D is positively correlated with the grade of glioma and the poor prognosis of gastric cancer (Ren et al., 2015; He et al., 2017).

### 7-Methylguanosine cluster in clear cell renal cell carcinoma

**FIGURE 2:**
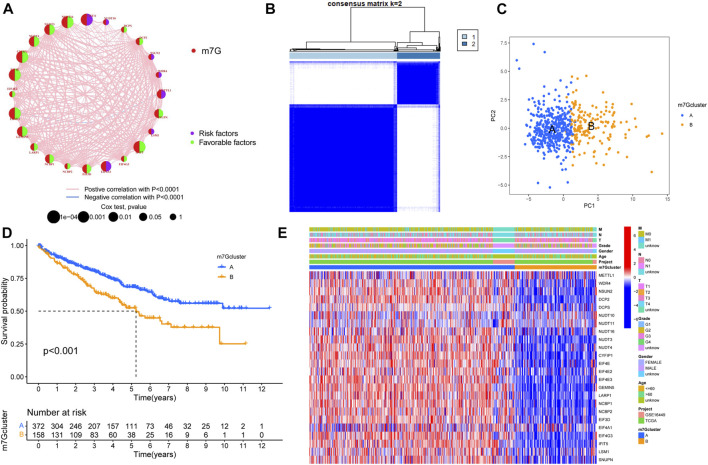
Characteristics of m7G subtypes. **(A)** The correlations between m7G-related genes; the line indicates that the two were related, the red line represented the positive correlation, the blue line represented the negative correlation, and the size of the node represented the prognostic correlation. **(B)** M7G-related genes were divided into two subtypes. **(C)** PCA analysis showed that there were significant differences among different subtypes. **(D)** Survival analysis of different subtypes. **(E)** The expression of m7G-related genes in different subtypes and the clinical characteristics of the subtypes. PCA, principal components analysis.

First, we merged the TPM data and the GSE16449 data using the “sva” package of R software for batch correction. Then we extracted the expression of m7G-related genes from all tumor samples. According to the univariate COX regression and Kaplan–Meier analysis, the prognosis-related m7G-related genes were found, and the m7G-related genes were divided into high- and low-risk groups, and the survival curve was plotted according to *p* < 0.05 (Supplementary Figure S1 and Supplementary Table S3). The prognosis network of m7G-related genes showed that the expression of most m7G-related genes was positively correlated; NUDT11, NUDT16, and CYFIP1 were the most likely genes related to prognosis (Figure 2A). We used K-mean method to cluster m7G-related genes, and K = 2 (Figure 2B) was the optimal value, and m7G-related genes were divided into two subtypes (m7Gcluster A *n* = 416 and m7Gcluster B *n* = 166). PCA showed that there were significant differences between the two m7Gclusters (Figure 2C). Figure 2D shows the survival rates between two different subtypes. There are significant differences between the two different subtypes, and the survival of m7Gcluster A is significantly better than that of m7Gcluster B. The heat map shows the differential expression of m7G-related genes between the two subtypes, as well as the difference between the two subtypes and clinical features. There were significant differences between the two subtypes, and most of the m7G-related genes were highly expressed in m7Gcluster A (Figure 2E).

### The correlations between different subtypes and the tumor immune microenvironment

**FIGURE 3:**
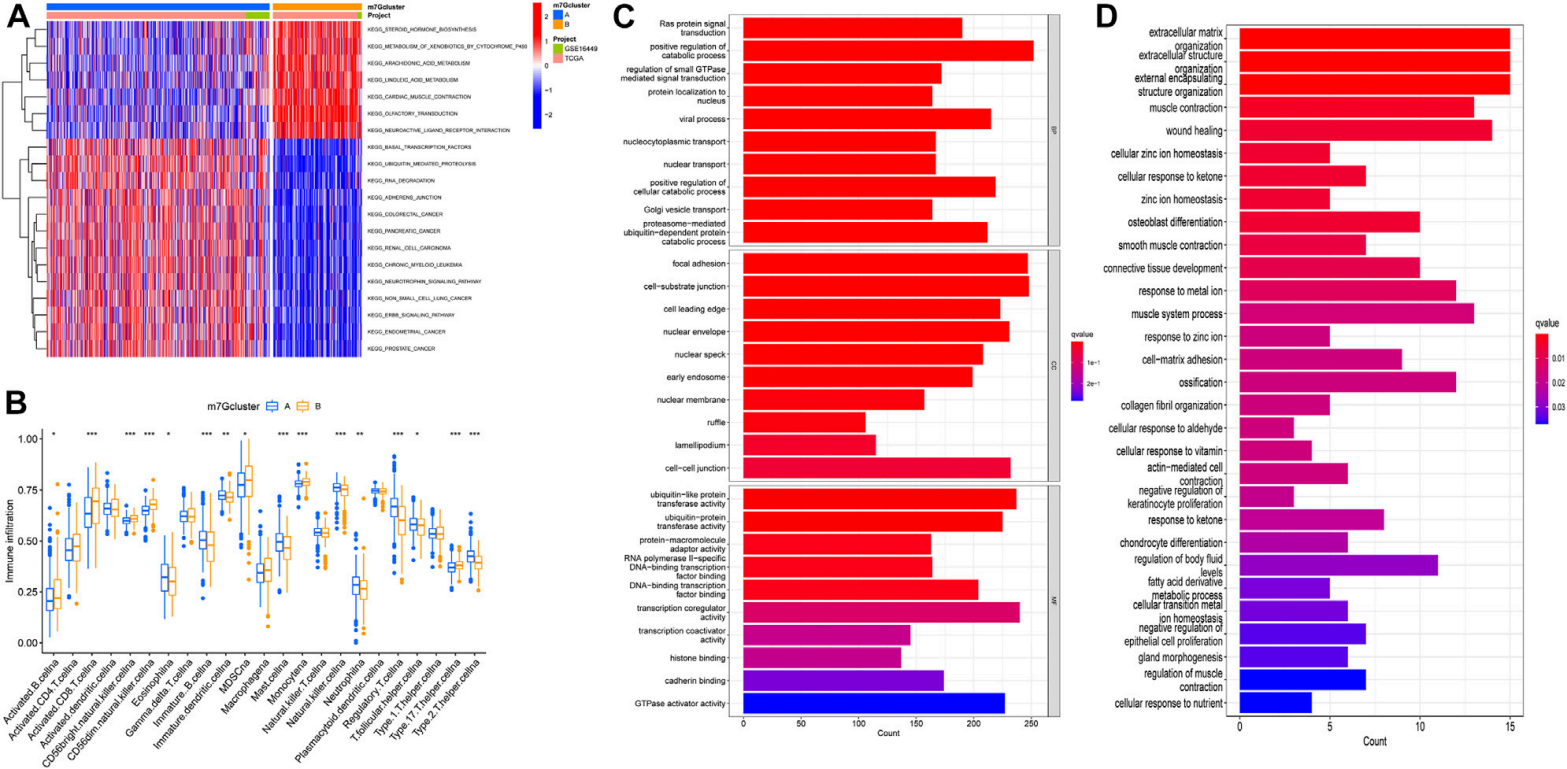
M7G subtype enrichment analysis. **(A)** GSVA enrichment analysis of different subtypes, blue and red represented inhibition and activation pathways, respectively. **(B)** Difference in immune infiltration of m7G subtypes. **(C,D)** GO and KEGG enrichment analysis. GSVA, gene set variation analysis; GO, Gene Ontology; KEGG, Kyoto Encyclopedia of Genes and Genomes.

GSVA enrichment analysis showed the enrichment pathway between different subtypes (Figure 3A). We can observe that pathways steroid hormone biosynthesis, metabolism of xenobiotics by cytochrome P450, arachidonic acid metabolism, linoleic acid metabolism, cardiac muscle contraction, olfactory transduction, and neuroactive ligand receptor interaction are active in m7Gcluster B, and other pathways are active in m7Gcluster A. Then we evaluated the difference between TIM and two m7Gclusters. Figure 3B shows the difference in the expression of 23 kinds of human immune cells and subtypes. All immune cells are significantly different between the two different subtypes, and the content of immune cells is higher in cluster B. We used “limma” package to screen the differentially expressed genes (DEG) related to m7G-related gene subtypes, and the adjusted *p* values of all the DEGs were less than 0.05. After that, the DEGs were analyzed by GO and KEGG enrichment analysis. Through GO enrichment analysis, we can observe that the DEGs are mainly enriched in biological processes and cell component, and the most significant one is positive regulation of the catabolic process (Figure 3C). KEGG enrichment analysis showed that the enrichment of DEGs were the most significant in extracellular matrix organization, extracellular structure organization, and external encapsulating structure organization (Figure 3D). These results suggest that m7G-related genes play an indispensable role in immune regulation.

### Screening of prognostic genes and construction of the model

**FIGURE 4:**
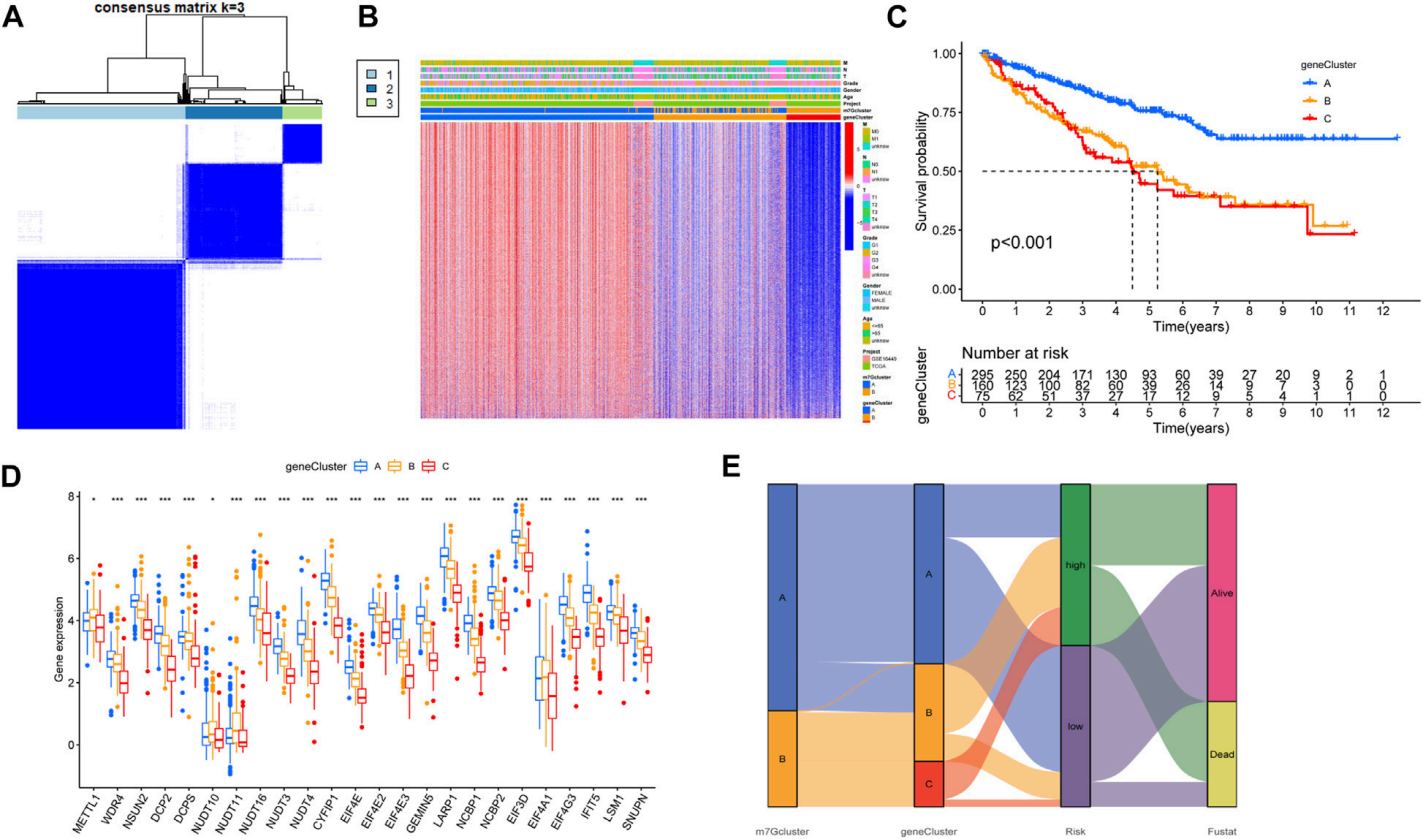
Characteristics of prognosis-related gene subtypes. **(A)** Prognosis-related genes were divided into three subtypes. **(B)** Survival analysis of prognosis-related gene subtypes. **(C)** The expression of prognosis-related gene subtypes and the clinical characteristics of the subtypes. **(D)** Correlations between prognosis-related gene subtypes and m7G-related genes. **(E)** The Sankey diagram in the process of constructing a prognostic model. *p < 0.05, **p < 0.01, ***p < 0.001.

According to the DEGs, univariate COX regression analysis (*p* < 0.05) was used to screen the prognosis-related genes (*n* = 4419). As with the m7G-related gene subtypes method, we divided the prognostic genes of ccRCC into three subtypes (Figure 4A). The survival analysis of the three subtypes showed that geneCluster A had more survival advantage than geneCluster B and C, and there were significant differences between the three subtypes (Figure 4B). The heat map showed the correlations between the three subtypes of prognostic and the clinical characteristics and m7G-related gene subtypes of ccRCC. Most of the genes were highly expressed in geneCluster A and lowly expressed in geneCluster C (Figure 4C). There is a significant difference between m7G-related genes and the two prognosis-related gene subtypes of ccRCC, which indicates that m7G-related genes are closely related to the prognosis of ccRCC (Figure 4D). The prognosis-related genes of ccRCC were analyzed by Lasso regression and multivariate Cox analysis (Supplementary Figure S2, Supplementary Table S4). Finally, seven genes were obtained. M7G Score = INPP4B * −0.3438 + PDK4 * −0.1936 + AJAP1 * −0.4170 + GADD45A * −0.3576 + IFI44 * 0.6223 + PPP1R1A * 0.1396 + HLA-DQB2 * −0.1989. The Sankey diagram showed the process of constructing the prognostic model (Figure 4E).

### Verification of the prognostic model

**FIGURE 5:**
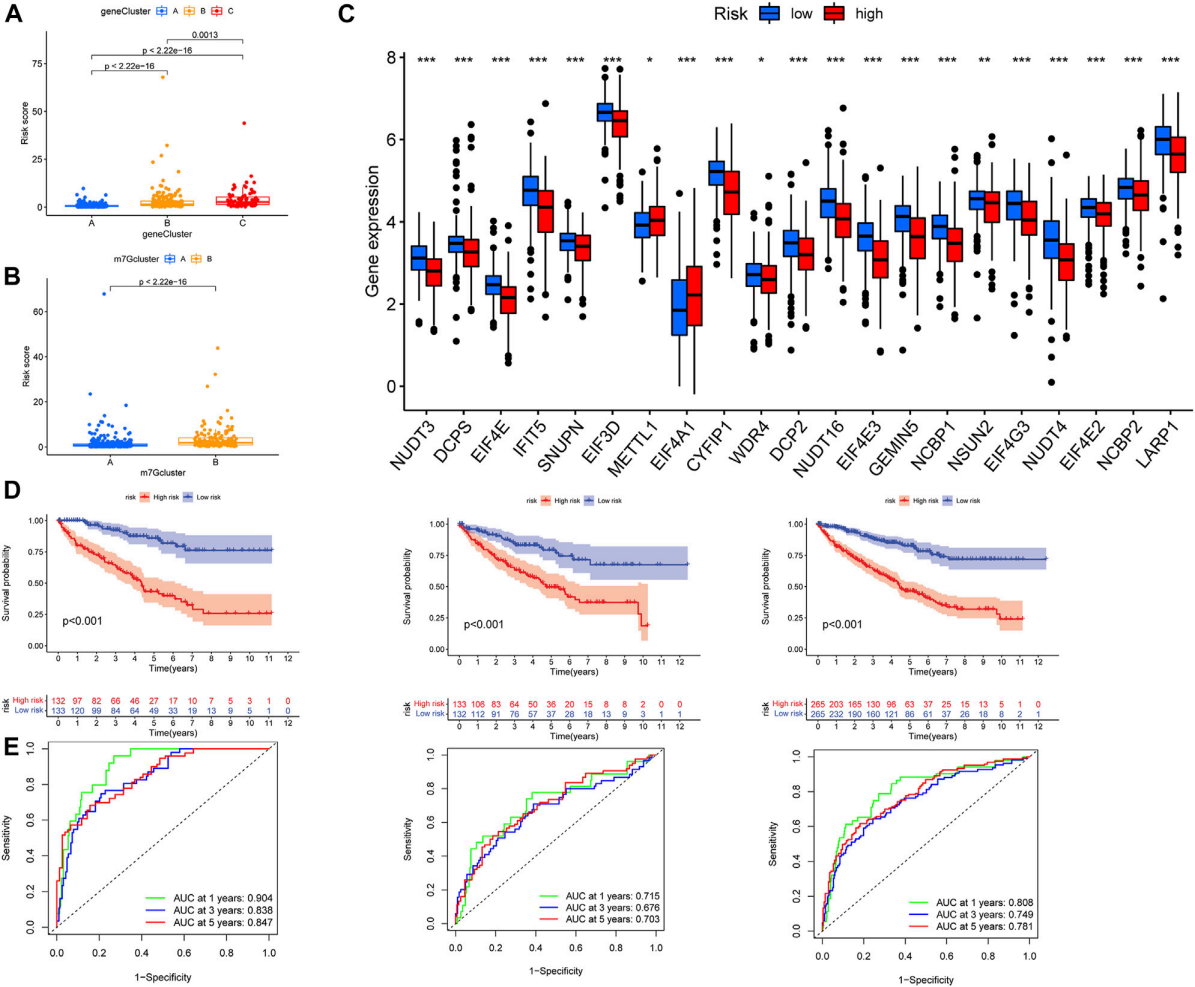
Verification of prognostic model. **(A)** Differential analysis of risk scores for prognosis-related gene subtypes. **(B)** Differential analysis of risk scores for m7G subtypes. **(C)** Expression difference in m7G-related genes in high- and low-risk groups. **(D)** From left to right, the patients in the train group, the test group, and all patients analyzed for survival. **(E)** From left to right, the patients in the train group, the test group, and all patients analyzed for ROC curves. ROC, receiver operating characteristic.

We observed the difference between the m7G score and the prognosis-related gene subtypes; Figure 5A shows that the geneCluster C score was the highest, and there was a significant difference between the three subtypes. There were also significant differences among m7G-related gene subtypes, and m7Gcluster B had higher m7G score (Figure 5B). The differential analysis of m7G-related genes between high- and low-risk groups showed that 21 m7G-related genes were significantly different between high- and low-risk groups, m7G-related genes were highly expressed in most low-risk groups, and METTL1 and EIF4A1 was highly expressed in high-risk groups (Figure 5C). Then we plotted the survival curves of the high- and low-risk patients and verified it with the test group. The results showed that there were significant differences between high- and low-risk patients in all groups, and the prognosis was better in the low-risk group (Figure 5D). In addition, the ROC curve of high- and low-risk patients in all groups had good validity (Figure 5E). The high-risk group had a poorer prognosis, METTL1 and EIF4A1 were highly expressed in the high-risk group, and these were consistent with our previous results, patients with high expression of METTL1 and EIF4A1 had a poorer prognosis, while the remaining m7G-related genes were the opposite (Supplementary Figure S1).

### Establishment of a nomogram

**FIGURE 6:**
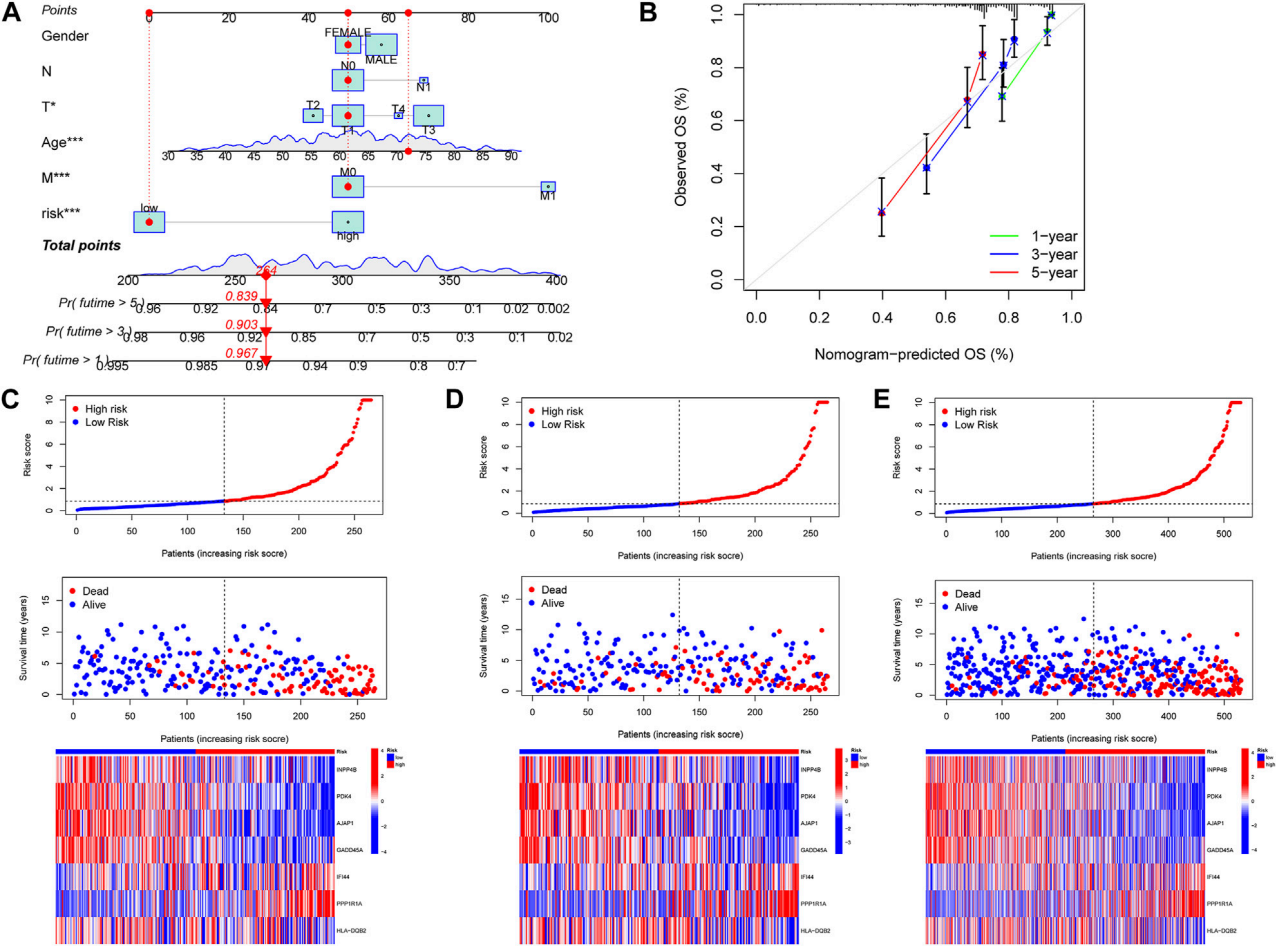
Construction of nomogram, the correlations between the m7G score and patient survival status. **(A)** Nomogram predicted 1, 3, and 5-year overall survival of ccRCC patients. **(B)** Calibration curves of the nomogram for predicting of 1-, 3-, and 5-year overall survival of ccRCC patients. **(C–E)** Distribution of the m7G score among ccRCC patients, correlations between the m7G score and patient survival status, risk heat map of m7G score genes, from left to right were the patients in the training group, the patients in the test group and the patients in all groups.

To easily and effectively predict the overall survival of ccRCC patients, we constructed a nomogram using the patient’s clinical information and the m7G score (Figure 6A). The results of the calibration curve show that the nomogram can effectively predict the 1-year, 3-year, and 5-year survival time of ccRCC patients (Figure 6B). The risk map shows the distribution of patients in high- and low-risk groups, and with the increase of the m7G score, the number of patients dying increases gradually; at the same time, we plotted a risk heat map, and the high-risk gene involved in the construction of the m7G score is IFI44 and PPP1R1A, the low-risk gene is INPP4B, PDK4, AJAP1, GADD45A, and HLA-DQB2 (Figures 6C–E).

### Correlations between 7-methylguanosine score and immune cells

**FIGURE 7:**
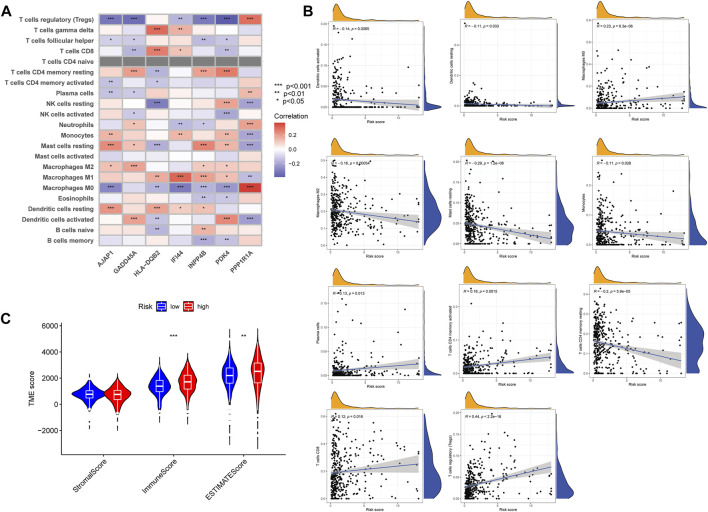
The correlations between the m7G score and TIM. **(A)** Correlations between m7G score genes and abundance of immune cells. **(B)** Correlations between the m7G score and immune cell types. **(C)** The correlations between high- and low-risk group patients and TIM. TIM, tumor immune microenvironment.

We analyzed the correlations between the seven genes and immune cells, and the results showed that there was a wide correlation between the m7G score and immune cells (Figure 7A). The correlation scatter plot showed that the m7G score was positively correlated with Macrophages M0, Plasma cells, T cells CD4 memory activated, T cells CD8, and T cells regulatory (Tregs) and negatively correlated with Dendritic cells activated, Dendritic cells resting, Macrophages M2, Mast cells resting, Monocytes, and T cells CD4 memory resting (Figure 7B). There were significant differences in high- and low-risk groups, the ImmuneScore and ESTIMATEScore were higher in the high-risk group (Figure 7C).

### Correlation between 7-methylguanosine score and clinicopathological features

**FIGURE 8:**
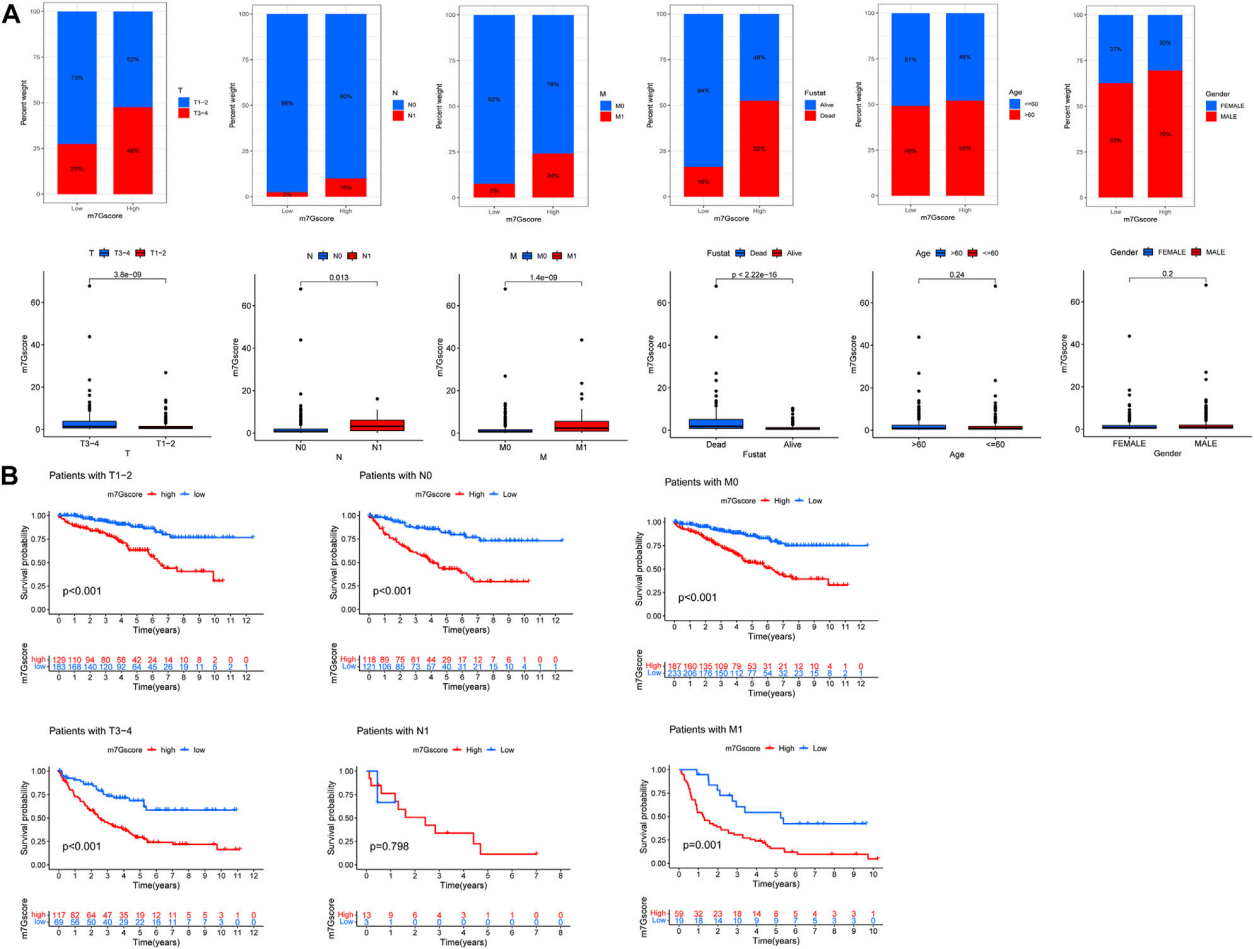
M7G score and ccRCC clinicopathological features. **(A)** Correlation between the m7G score and ccRCC clinicopathological features. **(B)** Correlation between the TNM stage survival and the M7G score.

We analyzed the correlation between the m7G score and clinicopathological features of ccRCC patients. The results showed that there was a significant correlation between TNM stages, survival status, and the m7G score, but the m7G score was not correlated with age and gender (Figure 8A). Higher m7G scores had higher TNM stage and death. In addition, there was a significant difference in the TNM stage between the high- and low-risk groups with m7G scores, and the patients in the low-risk group had better survival (Figure 8B).

### Mutation and immune checkpoints

**FIGURE 9:**
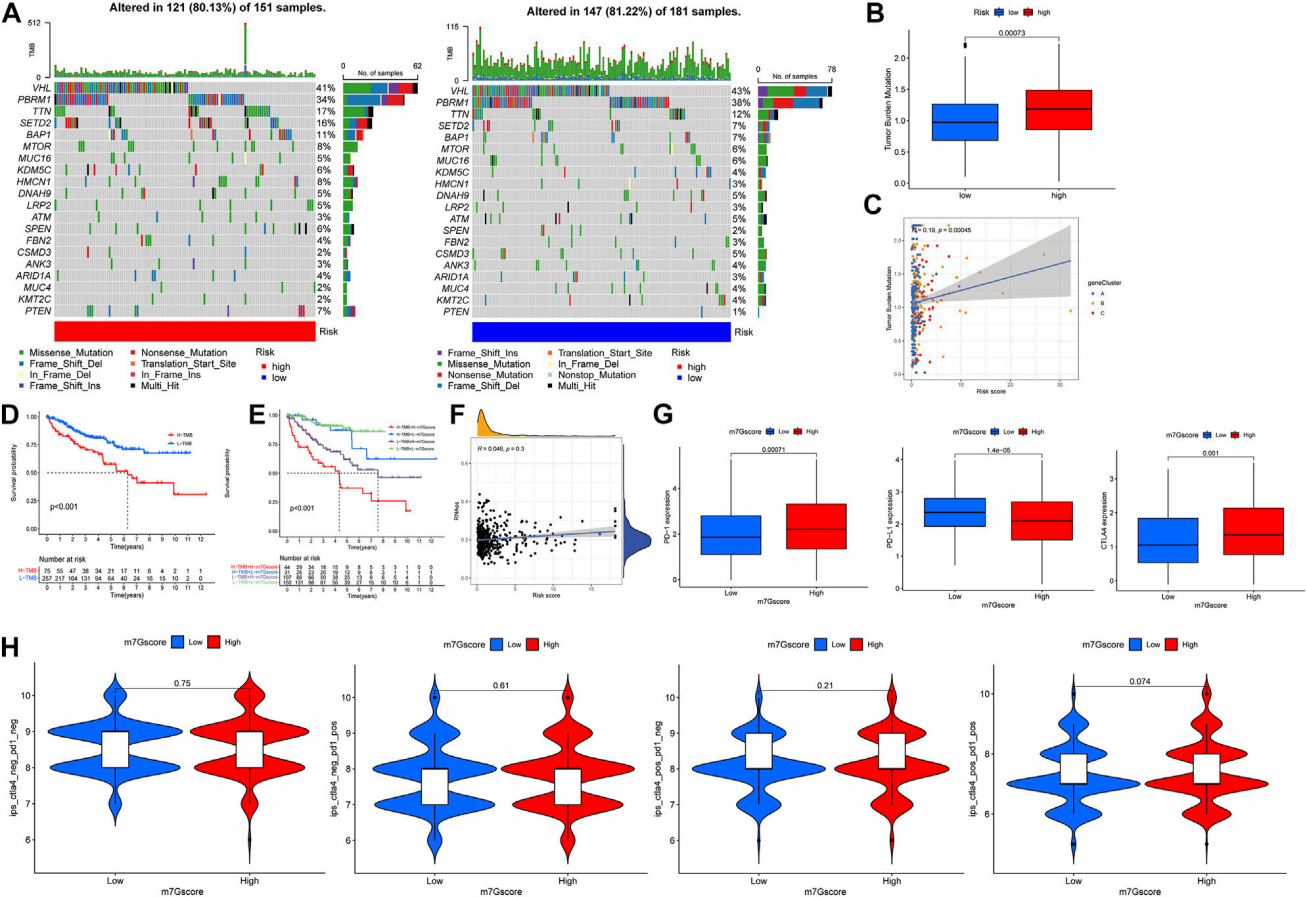
Mutation and immune checkpoint therapy. **(A)** Somatic mutation features of ccRCC patients in high- and low-risk groups, with colors representing different mutation types. **(B,C)** Correlations between the m7G score and tumor mutation burden. **(D,E)** Survival difference of patients with different TMB. **(F)** Correlations between the m7G score and RNAs. **(G)** Expression levels of PD-1, PD-L1 and CTLA4 in high- and low-risk groups. **(H)** Analysis of the m7G score in anti-PD-L1 and CTLA-4 immunotherapy.

The waterfall map shows the somatic mutation in the high- and low-risk groups, with the mutation rate of 83.13% in the high-risk group and 81.22% in the low-risk group. We showed the top 20 mutations in both groups, with the highest VHL mutation rate in the high- and the low-risk group (Figure 9A). In addition, the results of tumor mutational burden (TMB) showed that (Figures 9B,C) there was a significant difference between high- and low-risk groups; the high-risk group had a more significant TMB, and TMB positively correlates with the m7G score. There was a significant difference in survival between the high- and low-TMB groups, and the survival of patients with low TMB was significantly better than that of patients with high TMB, and the survival of patients with low TMB plus low m7G score was better (Figures 8D,E). The correlation analysis between the m7G score and stem cells showed that there was no significant correlation between the two (Figure 8F). In addition, our analysis of three important immune checkpoints showed that PD-1 and CTLA4 were highly expressed in the high-risk group while PD-L1 was lowly expressed (Figure 9G). Moreover, there was no significant difference between the four groups of immunotherapy in the high- and low-risk groups (Figure 9H). We further performed immunohistochemistry to externally verify the genes for METTL1, EIF3D, NUDT11, NUDT16, EIF4A1, IFI44, and CYFIP1 (https://www.proteinatlas.org/); the results showed that there were significant differences between ccRCC tissues and normal tissues (Supplementary Figure S3).

## Discussion

ccRCC is one of the most common tumors in urology, and the most effective treatment is still surgery; partial nephrectomy is the standard of surgical treatment for small renal mass (Campbell et al., 2021). About half of localized tumors have distant metastasis, and the prognosis of metastatic ccRCC patients is poor; ccRCC is the most common renal cell carcinoma (Gong et al., 2016; Navani and Heng, 2021). Immune checkpoint inhibitors have been used in clinic (Hu et al., 2021) and have achieved good results in a variety of tumor treatment (Arbour and Riely, 2019; O'Malley et al., 2021). Renal cell carcinoma is not sensitive to chemotherapy drugs, and immunotherapy and targeted therapy are effective treatments for advanced renal cell carcinoma (Bedke et al., 2021; Yoshida et al., 2021). Although advances in immunotherapy have been made in recent years, the heterogeneity of the tumor immune microenvironment often affects the progression and prognosis of renal cancer. We need new evidence to improve the current treatment.

M7G has a regulatory effect on the occurrence and development of tumors (Liu et al., 2019; Ma et al., 2021). Post-transcriptional modification of tRNA affects the structure and function of tRNA, thereby affecting the occurrence and development of a series of diseases. In the urinary system, a study (Ying et al., 2021) had shown that METTL1 is highly expressed in bladder cancer patients, and its expression level is positively correlated with the poor prognosis of patients. METTL1 affects the expression of some target genes by modifying the post-transcriptional modification of tRNA, thereby regulating tumors. At present, there are no studies on the correlations between m7G and the development of ccRCC, and the relationship between m7G-related genes and the immune microenvironment of ccRCC is unclear. So, multi-omics studies based on m7G-related genes are necessary. The m7G-related genes have different molecular regulation mechanisms among different tumors. Evaluation from multiple aspects can more effectively explain the role of m7G-related genes in the immune microenvironment of ccRCC, especially for personalized treatment and precision therapy.

In this study, we explored the expression patterns of m7G-related genes in ccRCC patients from multiple levels and analyzed the correlations between m7G-related genes and the ccRCC immune microenvironment, which paved the way for further discussions on how m7G-related genes can promote or inhibit TIM in the future. We had observed significant CNV in m7G-related genes. EIF4E1B, LARP1, GEMIN5, and DCP2 were the genes with the most significant increase in CNV. EIF4E1B is associated with eukaryotic canonical translation initiation; most eukaryotes have distinct EIF4E subgenes (Ying et al., 2021). LARP1 is involved in cell growth and proliferation, and its overexpression may reverse the progression of ccRCC [(Tcherkezian et al., 2014), (Li et al., 2020)]. GEMIN5 affects the assembly of motor neuron complexes; GEMIN5 mutations can shorten the life span of patients and affect movement and development (Kour et al., 2021). DCP2 affects human RNA stability and plays an important role in the regulation of immune responses (Luo et al., 2021). In our study, we found high expression of EIF4E1B, LARP1, GEMIN5, and DCP2 in tumor tissues, which seems to be consistent with our results. The role of m7G-related genes in ccRCC is worthy of further study. According to the expression of 29 m7G-related genes, we divided the ccRCC samples into two subtypes (m7Gcluster A and B) and identified them. Different subtypes showed different clinicopathological features and prognosis, and there were significant differences in TIM. There is higher m7G-related gene expression in m7Gcluster A, which is related to the higher stage and grade of ccRCC. We observed that there was a different immune cell infiltration in the two subtypes. Then we carried out GO, KEGG, and GSVA analyses to further explore the related functions and biological processes of the subtypes. With the study of TIM, the immune system is inextricably linked with the whole process of tumor progression (Kardoust Parizi et al., 2021).

Then, according to the differential expression of genes between the two subtypes, we divided the prognosis-related genes into three subtypes (geneCluster A, B, and C). There was a significant difference in survival between the three subtypes, and the prognosis of geneCluster A was better than geneCluster B and C. We also observed that most of the differential genes and m7G-related genes were highly expressed in subtype A, which was also associated with higher clinical stage and grade. Based on seven genes, we constructed a predictive model in the training group to evaluate ccRCC patients. We observed that most m7G-related genes were highly expressed in the low-risk group. In the train group and the test group, the overall survival rate of low-risk patients was significantly higher than high-risk patients, and the 1-, 3-, and 5-year AUC of the training group were 0.904, 0.838, and 0.847, respectively. Univariate and multivariate COX showed that the gene we used to construct the model was an independent prognostic factor—high expression of IFI44 and PPP1R1A in high-risk patients and high expression of INPP4B, PDK4, AJAP1, GADD45A, and HLA-DQB2 in low-risk patients. The study had shown that the expression level of IFI44 is associated with tyrosine kinase inhibitor resistance in non-small cell lung cancer (Wang S et al., 2020). Tumor metastasis often means worse prognosis. As a potential target of breast cancer, the expression level of PPP1R1A is closely related to the invasion and metastasis of breast cancer (Shi et al., 2020). We combined the m7G score and the clinical characteristics of ccRCC patients to create a nomogram, which gives full play to the advantages of the m7G score, which helps to objectively evaluate the prognosis of patients and better manage patients. One of the difficulties in tumor treatment is the lack of specificity. This study provides a new idea for the treatment of ccRCC and understands the molecular mechanism of ccRCC from many levels; our model may become a prognostic marker and potential therapeutic target for ccRCC. This has paved the way for subsequent targeted drug treatment.

We found that there were significant differences between the m7G score and immune cells. Macrophages vary with tumor progression (Biswas et al., 2008). In the initial stage, macrophages are often characterized by the accumulation of Macrophages M1. With the change of TIM, Macrophages M1 is gradually polarized to Macrophages M2. T cells CD8 plays an important role in host defense of the immune environment, and studies had shown that T cells CD8 infiltration is associated with poor prognosis in ccRCC [(Qi et al., 2020), (Murakami et al., 2021)]. Tregs are associated with renal cancer progression, and high expression of Tregs was detected in advanced ccRCC tissues (Minarik et al., 2013).

Significant mutations were found in the high- and low-risk groups of the m7G score, the highest mutation were VHL, and the proportion of missense mutation was the highest. VHL mutation is closely related to ccRCC. Low expression of PBRM1 and VHL is associated with increased invasiveness of ccRCC and may serve as a predictor of ccRCC growth rate (Hogner et al., 2018; Wang et al., 2021). We observed that there was a positive correlation between the m7G score and TMB, and there were significant differences between different risk groups and TMB. TMB may be related to TIM (Wang and Li, 2019). Zhang et al. (2019) compared the immune scores in the high- and low-TMB groups and found that there was a significant difference in immune cell infiltration between the two groups. This is consistent with our results, higher TMB in ccRCC may be associated with immune cell rejection. The m7G score we constructed can well predict the clinicopathological features of patients. Although the results showed that the m7G score did not predict survival in patients with N1 stage, it was fully explainable, and there were fewer patients with N1 stage and could not be effectively assessed.

At present, immune checkpoint blockade therapy (PD-1, PD-L1, and CTLA4) has been successfully applied to renal cancer patients with certain results (Kammerer-Jacquet et al., 2019; Tucker and Rini, 2020). In this study, we observed that there were significant differences in PD-1, PD-L1, and CTLA4 expression between high- and low-risk groups. Clinical trials have confirmed that PD-1 blockade can prolong the overall survival time of ccRCC patients, and ccRCC patients treated with nivolumab (anti-PD-1) are more effective than those treated with everolimus (Motzer et al., 2020a). Overall survival in ccRCC patients treated with nivolumab in combination with ipilimumab was better than sunitinib alone in the phase III CheckMate 214 trial (Motzer et al., 2020b). There was evidence that the progression-free survival of ccRCC patients treated with avelumab (anti-PD-L1) combined with axitinib is better than that of patients treated with sunitinib alone (Albiges et al., 2020; Tomita et al., 2020). To sum up, our model can predict the prognosis of ccRCC patients and the effect of immune checkpoint blockade therapy, which provides a new contribution to the immunotherapy of ccRCC.

This study has a good guiding role in the management of ccRCC patients, but it also has some limitations. Different case choices may affect the results of the study. Therefore, it is necessary to conduct a follow-up prospective study to verify it.

## Conclusion

There is a close relationship between m7G and ccRCC. The model we established from a multi-omics perspective provides new insights on the prognosis of ccRCC patients and further elaborates the relationship between ccRCC and TIM, which provides a new idea for immunotherapy.

## Data Availability

The datasets presented in this study can be found in online repositories. The names of the repository/repositories and accession number(s) can be found in the article/Supplementary Material.

## Supplementary material

Supplementary Figure S1 Correlations between the m7G-related genes and prognosis of clear cell renal cell carcinomas.

Supplementary Figure S2 Lasso regression of the prognostic model.

Supplementary Figure S3 Validation of gene expression results in tumor tissue and normal tissue by immunohistochemistry.
