# Supplementary figures and images for: Comprehensive analysis of 7-methylguanosine and immune microenvironment characteristics in clear cell renal cell carcinomas
Source: Front Genet. 2022 Aug 8;13:866819. doi: 10.3389/fgene.2022.866819 (PMC9393245; doi:10.3389/fgene.2022.866819)

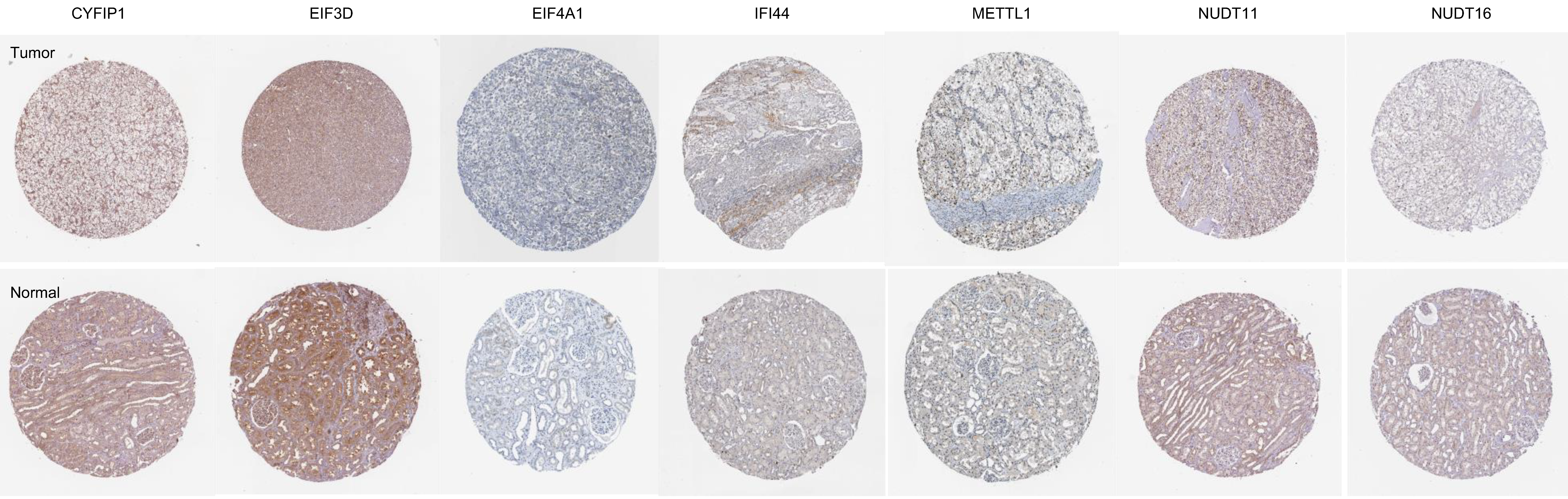

Supplement: Supplementary file 1 [file image3.tif]

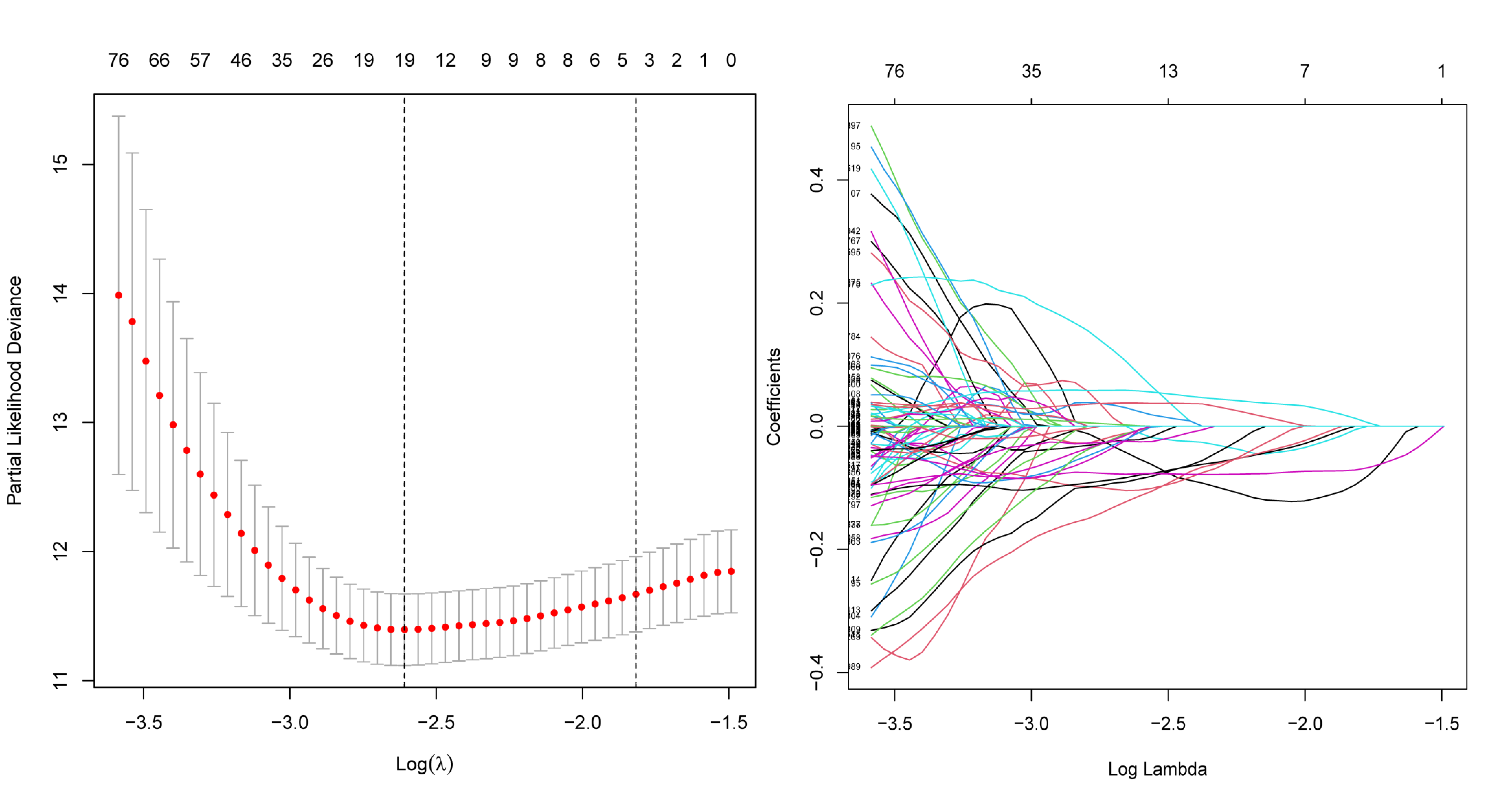

Supplement: Supplementary file 2 [file image2.tif]

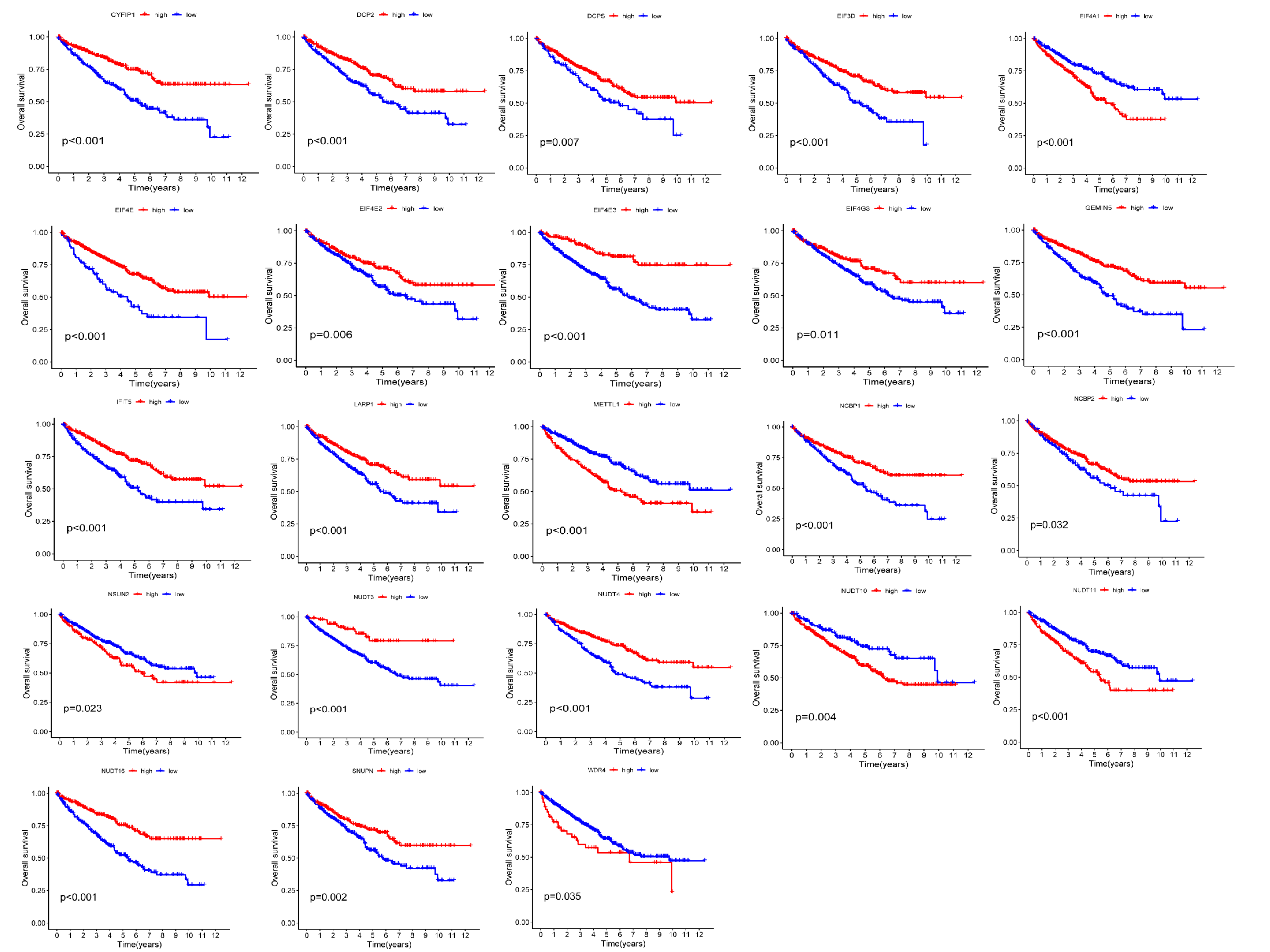

Supplement: Supplementary file 3 [file image1.tif]
