# Supplementary material for: Comprehensive analysis of 7-methylguanosine and immune microenvironment characteristics in clear cell renal cell carcinomas
Source: Front Genet. 2022 Aug 8;13:866819. doi: 10.3389/fgene.2022.866819 (PMC9393245; doi:10.3389/fgene.2022.866819)
Supplement: Supplementary file 4 [file Datasheet1.docx]

**TABLE. S1 Clinical Information.**

| Covariates | Type | Number=607 |
| --- | --- | --- |
| Age | <=60 | 266 (43.8%) |
| Age | >60 | 271 (44.7%) |
| Age | unknow | 70.(11.5%) |
| Gender | female | 191 (19.6%) |
| Gender | male | 346 (68.9%) |
| Gender | unknow | 70.(11.5%) |
| Grade | Grade 1 | 14 (2.2%) |
| Grade | Grade 2 | 230 (37.9%) |
| Grade | Grade 3 | 207 (34.1%) |
| Grade | Grade 4 | 78 (12.9%) |
| Grade | unknow | 78 (12.9%) |
| T | T1 | 275 (45.3%) |
| T | T2 | 69 (11.4%) |
| T | T3 | 182 (30.0%) |
| T | T4 | 11 (1.8%) |
| T | unknow | 70.(11.5%) |
| M | M0 | 426 (70.2%) |
| M | M1 | 79 (13.0%) |
| M | unknow | 102 (16.8%) |
| N | N0 | 240 (39.5%) |
| N | N1 | 17 (2.8%) |
| N | unknow | 350 (57.7%) |

**TABLE. S2 m7G-related genes.**

| Gene | Type |
| --- | --- |
| METTL1 | m7G |
| WDR4 | m7G |
| NSUN2 | m7G |
| DCP2 | m7G |
| DCPS | m7G |
| NUDT10 | m7G |
| NUDT11 | m7G |
| NUDT16 | m7G |
| NUDT3 | m7G |
| NUDT4 | m7G |
| NUDT4B | m7G |
| AGO2 | m7G |
| CYFIP1 | m7G |
| EIF4E | m7G |
| EIF4E1B | m7G |
| EIF4E2 | m7G |
| EIF4E3 | m7G |
| GEMIN5 | m7G |
| LARP1 | m7G |
| NCBP1 | m7G |
| NCBP2 | m7G |
| NCBP3 | m7G |
| EIF3D | m7G |
| EIF4A1 | m7G |
| EIF4G3 | m7G |
| IFIT5 | m7G |
| LSM1 | m7G |
| NCBP2L | m7G |
| SNUPN | m7G |

**TABLE. S3 Univariate Cox Result.**

| id | HR | HR.95L | HR.95H | pvalue | km |
| --- | --- | --- | --- | --- | --- |
| METTL1 | 1.543454 | 1.131726 | 2.10497 | 0.006114 | 2.08E-05 |
| WDR4 | 1.12552 | 0.8403 | 1.507553 | 0.42776 | 0.034748 |
| NSUN2 | 1.055506 | 0.786604 | 1.416333 | 0.718797 | 0.023384 |
| DCP2 | 0.78299 | 0.618503 | 0.99122 | 0.042027 | 0.000245 |
| DCPS | 0.801571 | 0.596332 | 1.077448 | 0.142741 | 0.007497 |
| NUDT10 | 1.169014 | 0.937652 | 1.457464 | 0.165187 | 0.003994 |
| NUDT11 | 1.667385 | 1.403625 | 1.980708 | 5.92E-09 | 0.000152 |
| NUDT16 | 0.617366 | 0.489849 | 0.778079 | 4.40E-05 | 7.10E-08 |
| NUDT3 | 0.591486 | 0.44086 | 0.793575 | 0.000462 | 0.000336 |
| NUDT4 | 0.697534 | 0.575821 | 0.844974 | 0.000232 | 7.53E-06 |
| CYFIP1 | 0.612427 | 0.497458 | 0.753967 | 3.80E-06 | 1.69E-07 |
| EIF4E | 0.54052 | 0.397699 | 0.734631 | 8.50E-05 | 6.96E-06 |
| EIF4E2 | 0.81997 | 0.60099 | 1.118738 | 0.210516 | 0.005849 |
| EIF4E3 | 0.594436 | 0.491456 | 0.718995 | 8.38E-08 | 1.94E-07 |
| GEMIN5 | 0.675071 | 0.550758 | 0.827444 | 0.000154 | 9.45E-07 |
| LARP1 | 0.760205 | 0.615756 | 0.938539 | 0.010775 | 0.000519 |
| NCBP1 | 0.682975 | 0.536181 | 0.869957 | 0.002013 | 7.09E-05 |
| NCBP2 | 0.961138 | 0.706406 | 1.307727 | 0.800817 | 0.032231 |
| EIF3D | 0.669384 | 0.49347 | 0.90801 | 0.009872 | 8.10E-05 |
| EIF4A1 | 1.310591 | 1.128264 | 1.522381 | 0.000402 | 9.83E-05 |
| EIF4G3 | 0.854674 | 0.674094 | 1.083628 | 0.194717 | 0.011219 |
| IFIT5 | 0.6512 | 0.530354 | 0.799583 | 4.21E-05 | 8.28E-06 |
| LSM1 | 1.03769 | 0.744487 | 1.446366 | 0.827136 | 0.057419 |
| SNUPN | 0.559557 | 0.3895 | 0.803862 | 0.001683 | 0.001907 |

**TABLE. S4 Multivariate Cox Result.**

| id | coef |
| --- | --- |
| INPP4B | -0.343761765 |
| PDK4 | -0.193614028 |
| AJAP1 | -0.416996691 |
| GADD45A | -0.357636409 |
| IFI44 | 0.622324281 |
| PPP1R1A | 0.139561232 |
| HLA-DQB2 | -0.198850835 |
